# Colloidal Synthesis and Characterization of Molybdenum Chalcogenide Quantum Dots Using a Two-Source Precursor Pathway for Photovoltaic Applications

**DOI:** 10.3390/molecules26144191

**Published:** 2021-07-09

**Authors:** Evernice Chikukwa, Edson Meyer, Johannes Mbese, Nyengerai Zingwe

**Affiliations:** 1 Fort Hare Institute of Technology (FHIT), Private Bag X1314, Alice 5700, South Africa; 201515550@ufh.ac.za (E.C.); emeyer@ufh.ac.za (E.M.); 2 Department of Chemistry, University of Fort Hare, Alice 5700, South Africa; jmbese@ufh.ac.za; 3 Energy, Materials and Inorganic Chemistry Research Group (EMICREG), University of Fort Hare, Alice 5700, South Africa

**Keywords:** chalcogenides, metals, chemical synthesis

## Abstract

The drawbacks of utilizing nonrenewable energy have quickened innovative work on practical sustainable power sources (photovoltaics) because of their provision of a better-preserved decent environment which is free from natural contamination and commotion. Herein, the synthesis, characterization, and application of Mo chalcogenide nanoparticles (NP) as alternative sources in the absorber layer of QDSSCs is discussed. The successful synthesis of the NP was confirmed as the results from the diffractive peaks obtained from XRD which were positive and agreed in comparison with the standard. The diffractive peaks were shown in the planes (100), (002), (100), and (105) for the MoS_2_ nanoparticles; (002), (100), (103), and (110) for the MoSe_2_ nanoparticles; and (0002), (0004), (103), as well as (0006) for the MoTe_2_ nanoparticles. MoSe_2_ presented the smallest size of the nanoparticles, followed by MoTe_2_ and, lastly, by MoS_2_. These results agreed with the results obtained using SEM analysis. For the optical properties of the nanoparticles, UV–Vis and PL were used. The shift of the peaks from the red shift (600 nm) to the blue shift (270–275 nm and 287–289 nm (UV–Vis)) confirmed that the nanoparticles were quantum-confined. The application of the MoX_2_ NPs in QDSSCs was performed, with MoSe_2_ presenting the greatest PCE of 7.86%, followed by MoTe_2_ (6.93%) and, lastly, by MoS_2_, with the PCE of 6.05%.

## 1. Introduction

Fossil fuels such as coal, oil, and natural gases deliver approximately 80% of the whole world’s energy. Energy generated from nonrenewable energy sources has a drawback of prompted outflow of ozone-harming substances. The emission of greenhouse gases has an effect on the environment as it leads to its degradation as well as to global warming. These drawbacks have quickened innovative work of practical sustainable power sources because of their provision of a better-preserved environment free from natural contamination and commotion. The primary forms of sustainable energy which could potentially be of economic value as suitable alternatives to nonrenewable energy are bioenergy, solar energy photovoltaics, and wind power. The harnessing of solar energy into electricity or other forms of power is conducted with photovoltaic systems and instruments. Solar-based power is utilized on account of its low ecological effect and as an ideal possibility for elective energy sources because of its global accessibility [1,2].

The most readily accessible solar cells are silicon-based solar-powered cells which are the most effective in converting sunlight into electricity. They have been the most effective because they have a strong optical absorption due to sp antibonding coupling, high electron–hole mobilities, and diffusion lengths. Nevertheless, silicon-based solar cells also have disadvantages including an intensive and expensive process of purifying silicon to be used in the solar cells as well as lower efficiency [3,4,5].

A few methodologies have been adopted so as to have solar-powered cells that are competent to overcome the market share of nonrenewable energy sources such as coal and oil. Quantum dots were conjectured during the 1970s and first made in the mid-1980s and have demonstrated effective features that can improve the properties of solar cells. If semiconductor particles are minute, quantum effects become possibly the most important factor, which limit the energies at which electrons and holes can exist in particles. As energy is identified by wavelength, optical properties of a molecule can be fine-tuned relying upon its size. Therefore, particles can be made to transmit or absorb explicit wavelengths of light just by controlling their size [6]. Quantum dots are artificial nanostructures that can have many shifted properties, contingent upon their material and shape. They can be utilized as dynamic materials in single-electron transistors because of their specific electronic properties. The properties of a quantum dot are dictated by its size as well as by its shape, composition, and structure. Quantum dots have found wide use in catalysis, gadgets, photonics, data stockpiling, imaging, medication, or detection [1,6,7].

In solar cells, quantum-confined semiconductor nanocrystals have incredible optical, electronic, and physical properties which further enhance electron generation and transportation around the circuit. Quantum dots (QD) are also much more efficient conductors than their bulk material, and they reduce recombination losses. Their band gap can be designed by manipulating their size and shape. Introducing nanoparticles in the absorber layer of the DSSC enhances the effective surface area and subsequently the dye adsorption [8,9]. A study of new materials with new properties might be favorable in improving the function of photovoltaic devices and utilizing the related innovations. Molybdenum can exhibit very good stability when heated at higher temperatures and allows for good adhesion of the active layer compared to other quantum dots. Therefore, this study focuses on the synthesis and characterization of molybdenum dichalcogenide quantum dots from two-source precursors for application in photovoltaics [10,11].

## 2. Results

### 2.1. XRD Analysis

**Figure 1:**
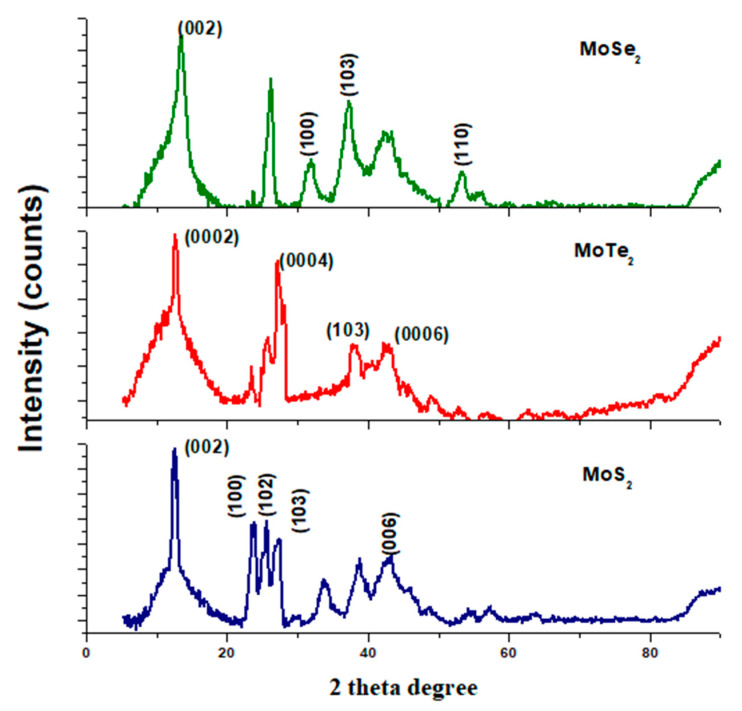
XRD spectra for the synthesized MoX_2_ nanomaterials.

The crystallographic properties of the synthesized metal chalcogenides were determined using X-ray diffraction analysis which was performed at room temperature. Standard hexagonal 2H–MoS_2_ (JCPDS No. 37-1492), hexagonal 2H–MoSe_2_ (JCPDS No. 29-0914), and 2H–MoTe_2_ (JCPDS No. 01-072-011) were used, and the diffraction patterns are shown in Figure 1 below [12,13,14]. The patterns obtained from all the molybdenum chalcogenides were slightly different from the standard hexagonal plane of MoX_2_ (X = S, Se, Te). The sharp peaks obtained denote that the products obtained were crystalline.

Nanocrystals from MoS_2_ and MoSe_2_ had the most significantly intense peaks at 12.8° and 13.8°, respectively, and these were both oriented in the (002) plane. This higher intensity of the peaks signifies that the nanocrystals of MoSe_2_ and MoS_2_ were of high crystallinity. The sample of MoTe_2_ exhibited diffraction peaks that were almost similar to the planes (0002), (0004), and (0006) attributed to MoTe_2_ being perpendicularly oriented to the film plane as shown in Figure 1 and Table 1 [13]. The widening of the XRD peaks observed in Figure 1 for MoSe_2_ and MoS_2_ might be due to some stacking faults and structural disorder, hence the widened peaks. Some of the peaks observed did not correspond to the JCPDS reference standards used; the presence of those peaks might be due to some impurities present in the nanomaterials.

The Debye–Scherrer equation (Equation (1)) was used to determine the crystallite size from the half-width of the diffraction peaks [15,16].

D = (αλ)/βcosθ (1)

where D is the mean particle size; α is the geometric factor (0.94); λ is the x-ray wavelength (1.5406 Å); β is the full width at half maximum (FHWM); θ is the angle of the diffraction peak.

The mean particle sizes were calculated, and the values are shown in Table 2; MoSe_2_ had the smallest particle size, followed by MoTe_2_ and, lastly, by MoS_2_. The peak width varied with the crystallite size, although indirectly: the higher the FWHM, the smaller the crystallite size.

### 2.2. SEM Analysis

**Figure 2:**
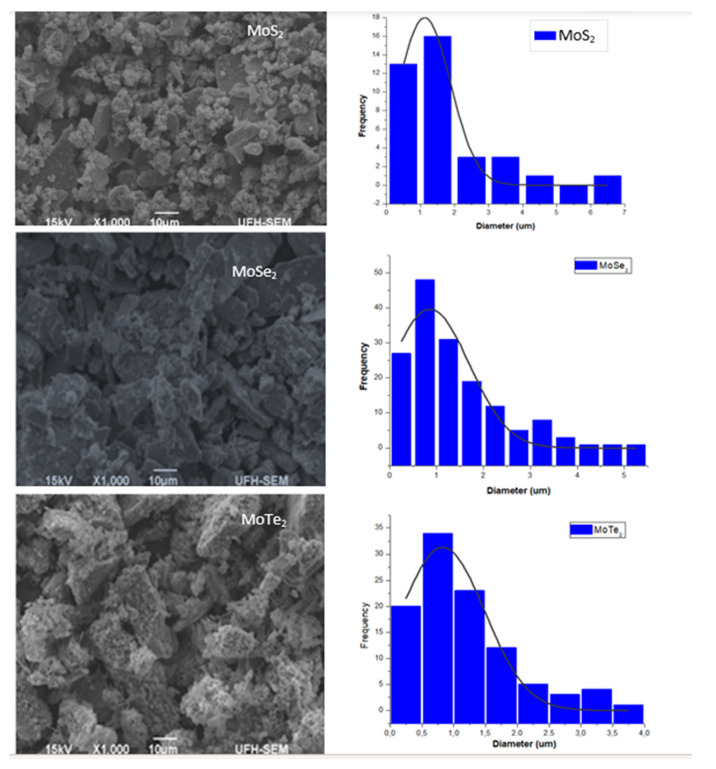
SEM images and grain size distribution for the molybdenum chalcogenides.

Scanning electron microscopy (SEM) was utilized to analyze the morphology and grain sizes of the synthesized nanoparticles. Figure 2 shows the images obtained by SEM analysis as well as the particle size distribution. The sample containing the as-synthesized MoS_2_ showed some dispersion of the nanoparticles with some clusters. A higher degree of aggregation and clustering was also observed in MoTe_2_ as shown in the SEM images. The MoSe_2_ nanoparticles were agglomerated as shown in Figure 2. These images are similar to the images published by Zhang et al. in 2017 [15]. The grain size distribution of the nanoparticles was evaluated as well using the results obtained from SEM images using the Image J application (Image J 1.50i, Java 1.8.0_77, Wayne Rasband, National Institute of Health, Bethesda, MA, USA) and Origin 6.1 (V6.1052(B232), OriginLab Cooperation, MA, USA). Table 3 shows the mean particle sizes obtained.

From the results obtained, it can be deduced that MoSe_2_ had the smallest grain size with an average diameter of 0.81718 µm, followed by MoTe_2_ with 0.82577 µm and, lastly, by MoS_2_ with the mean grain size of 1.122 µm. The particle sizes of the MoX_2_ NPs could only be calculated using XRD results, and the particle size of all the nanomaterials fell within the quantum dot nanoparticle range. Only the grain sizes of the agglomerated nanoparticles could be obtained using SEM analysis as it has a lower resolution.

### 2.3. FTIR Analysis of MoX_2_ Nanoparticles

**Figure 3:**
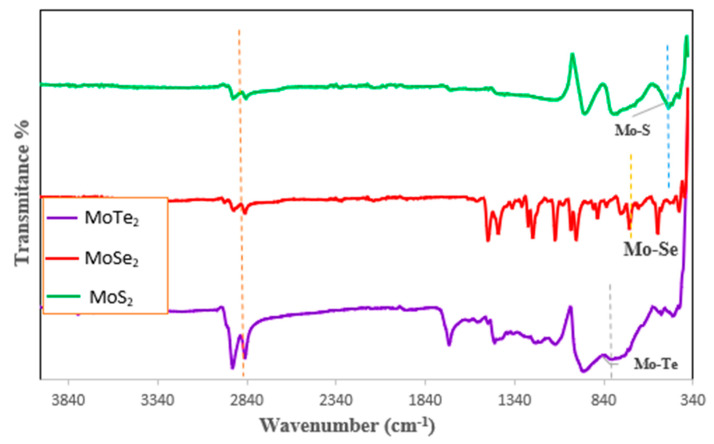
FTIR spectra for the MoX_2_ nanoparticles synthesized from two-source precursors.

The synthesized MoX_2_ nanoparticles were infrared-active; hence, only FTIR was used to discuss the confirmation of the structure of the synthesized nanomaterials. The successful synthesis of the nanoparticles was confirmed by the availability of the Mo–X peaks in all the three graphs shown in Figure 3. The Mo–Te vibration band was observed at 812 cm^−1^, Mo–Se—at 689 cm^−1^, Mo–S—at 459 cm^−1^. The peaks of the Mo–X stretch observed in these nanomaterials agreed with what is found in the literature [9,11,17]. A C–H stretch was observed at 2920 cm^−1^ which was attributed to the capping agent TOPO that was used. The other peaks shown in the graphs were due to the composition of the capping agent that was used as well.

### 2.4. Optical Properties of the MoX_2_ Nanoparticles

**Figure 4:**
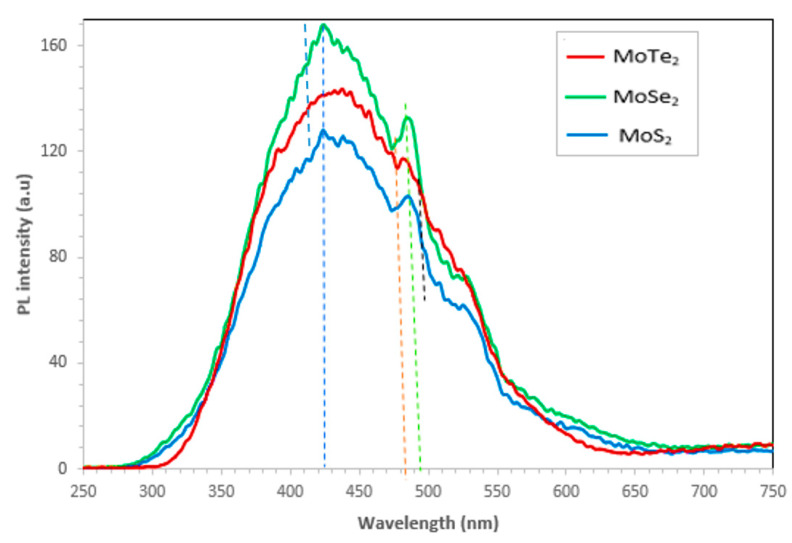
Emission spectra of the MoX_2_ (X = S, Se, Te) nanoparticles.

**Figure 5:**
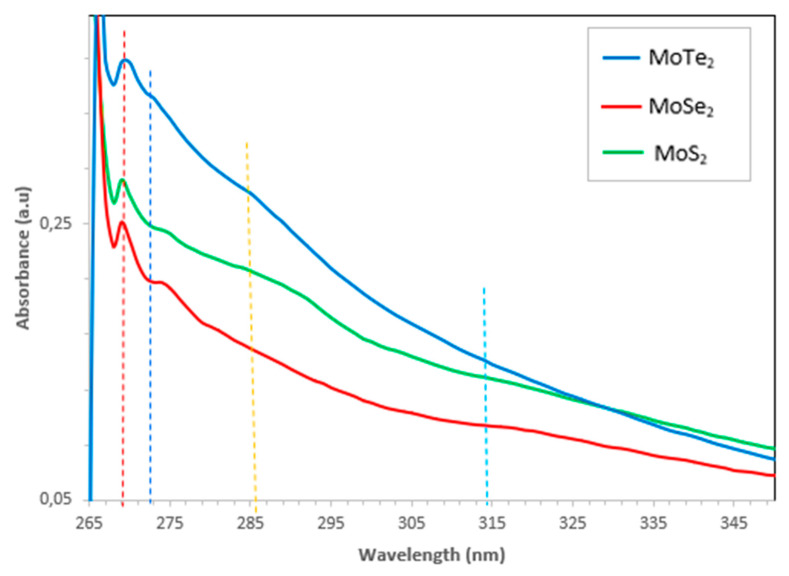
UV–Vis spectra of the MoX_2_ (X = S, Se, Te) nanoparticles.

The optical properties of the synthesized Mo chalcogenide nanoparticles were studied using ultraviolet–visible spectroscopy (UV–Vis) and photoluminescence (PL) spectroscopy. Figure 4 and Figure 5 show results of the UV–Vis and PL analyses. The nanomaterials were dissolved in a solution to perform the analysis.

Photoluminescence spectra results for the Mo chalcogenide nanoparticles are shown in Figure 4. The most intense peak was observed for all the MoX_2_ nanoparticles at 432 nm, signifying the quantum confinement of MoX_2_. MoSe_2_ exhibited the highest PL intensity followed by MoTe_2_ and, lastly, by MoS_2_; this is attributed to the aggregation and dispersion of the nanoparticles. Hence, high intensity for MoSe_2_ reveals that the nanoparticles were dispersed, whereas the MoS_2_ nanoparticles were aggregated. This is further confirmed by the SEM images obtained in Figure 2. Another peak was also observed at 488 nm for all the nanoparticles. Another peak was also exhibited on the spectra for only MoS_2_ and MoSe_2_ at 534 nm; this was ascribed to the recombination of holes or electrons that were trapped in surface defect state of the MoX_2_ nanoparticles [13]. In 2014, Amara et al. recorded a peak of 662 nm for bulk MoS_2_ [18]; thus, a shift to 488 nm of MoS_2_ symbolized the synthesis of a monolayer of MoS_2_ which is a semiconductor with a direct bandgap of approximately 1.92 eV. The photoluminescence characteristics of transition metal chalcogenides can be influenced by the type of solvents used, defects, type of substrate used, intensity of laser excitation, chemical doping, molecular physisorption, as well as heterostructuring [19,20,21].

UV–Vis spectroscopy was used to study the optical properties of the synthesized molybdenum chalcogenide nanoparticles. Basically, the nanoparticle size is directly proportional to the wavelength at the maximum exciton absorption (λ_max_); hence, as λ_max_ decreases, the nanoparticle size also decreases; this is a result of the photogenerated electron–hole carriers’ quantum confinements. The spectra of the MoX_2_ are shown in Figure 5 above. Distinctive peaks were observed at 270 nm and 275 nm for all the MoX_2_ nanoparticles synthesized. Another broad and very shallow peak was again observed at 287–289 nm; this peak was only visible in all the MoS_2_ and MoTe_2_ nanoparticles. MoTe_2_ and MoSe_2_ presented a very shallow peak at 302–322 nm as shown in the insert. The peaks around 302–322 nm were ascribed to the optical transitions flanked by the state peak density in the conduction and valence bands. The blue shift of the absorption peak clearly indicated that the synthesized MoX_2_ nanoparticles were quantum-confined. The optical transitions of the synthesized MoX_2_ nanoparticles were remarkably different from the bulk MoX_2_. This was further explained by the absence of the K point of the Brillouin zone which typically shows a peak at 615–670 nm. The absence of this peak was due to quantum confinement effects as well as the quasi-incessant electronic energy [17].

### 2.5. Current–Voltage and Photovoltaic Measurements

**Figure 6:**
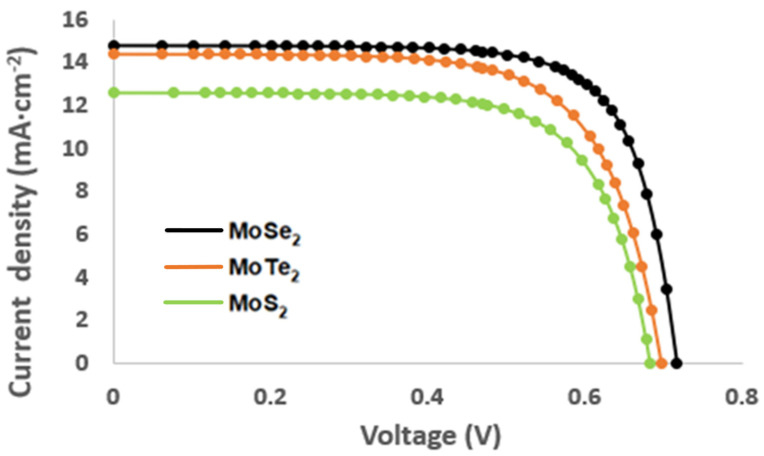
Current–voltage characteristic of the MoX_2_ (X = S, Se, Te) nanoparticles.

The current–voltage characteristics of the developed solar cells utilizing the synthesized chalcogenides as the sensitizer are depicted in Figure 6 and Table 4 below. The efficiency of the developed QDSSCs fabricated from the Mo chalcogenides was calculated using the photovoltaic measurements taken as shown in Figure 6 below. The fill factor and efficiency of the developed cells were calculated using Equations (2) and (3) below:

PCE = (P_MAX_/P_IN_) × 100 (2)

FF = (P_MAX_/V_OC_ × I_SC_) (3)

where P_MAX_ is the maximum power and P_IN_ is the incident power, V_OC_ is the open circuit voltage and I_SC_ is the short circuit current.

Power conversion efficiency for the developed solar cells increased in the following order: MoS_2_ (6.05%) < MoTe_2_ (6.93%) < MoSe_2_ (7.05%). MoS_2_ exhibited the lowest PCE; this was due to the nanoparticles having a higher charge transfer resistance, which resulted in the limitation of electron transfer, subsequently resulting in low electron–hole recombination, hence, in limited efficiency [22]. Low efficiency might be due to the increased surface area and thickness on the photoanode which results in charge transfer resistance [23,24]. Furthermore, according to the chemical composition of the samples, the fill factor (FF) was used to determine the quality of the fabricated QDSSCs. MoSe_2_ demonstrated the best quality of the cell as it had an FF of 73.7%, followed by MoS_2_ that had an FF of 70.6% and, lastly, by MoTe_2_ with an FF of 68%. The variation in the FF can be attributed to higher rates of electron–hole recombination stemming from increased charge transfer resistance which induces lower carrier mobilities leading to a reduced fill factor.

## 3. Materials and Methods

The chemicals utilized in the synthesis procedure were molybdenum (IV) oxide (Sigma-Aldrich, St Louis, MO, USA, 99.5%); selenium powder (Sigma-Aldrich, St Louis, MO, USA, 99.9%); monoclinic sulfur (Saarchem, Mumbai, India, 98–102%); tellurium dioxide (Sigma-Aldrich St Louis, MO, USA, 99%); oleic acid (Sigma-Aldrich St Louis, MO, USA, 90%); tri-n-octylphosphine oxide (TOPO) (Merck KGaA, Modderfontein, South Africa, 98%).

### 3.1. Synthesis of the MoX_2_ Nanoparticles from Two-Source Precursors

The synthesis procedure for ligands and complexes conducted in this work was adopted and modified from the literature and the experiments conducted by Mbese et al., Agoro et al., Meyer et al. [24,25,26], as well as Halimehjane [27]. As an example, to explain the synthesis process, the procedure utilized to synthesize MoS_2_ was explained. In the solvent (4 mL oleic acid), 2.877 g of MoO_2_ (0.02 mol) were dissolved. To create a homogenous mixture, the solution was mixed for some time, approximately 10 min, before being transferred to the colloidal setup. In 4 mL of oleic acid, 0.6412 g of sulfur (chalcogenide source, 0.02) was also dissolved and heated with continuous stirring until a homogenous mixture was reached. Two-source precursors were then added to hot TOPO (capping agent) and the temperature was kept constant at 360 °C for an hour. The reaction was then left to cool down to approximately 70 °C; then, ethanol was added to precipitate the nanoparticles. After precipitation, the product was centrifuged at 3500 rpm for 15 min thrice. Toluene was then added to disperse the synthesized nanoparticles.

**Figure 7:**
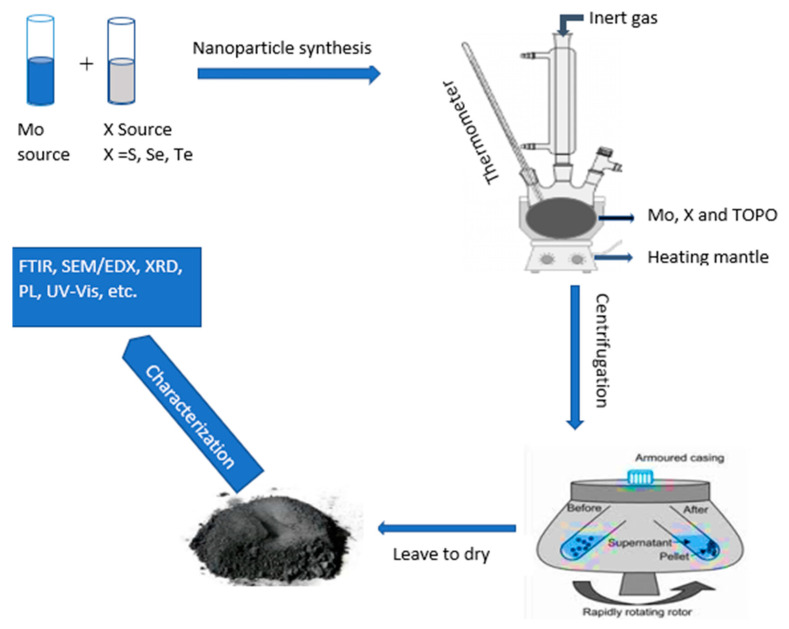
Synthesis procedure for the molybdenum chalcogenide nanoparticles from two-source precursors.

The same procedure was used for the synthesis of MoSe_2_ and MoTe_2_ with Se powder used as a Se source for MoSe_2_ synthesis and tellurium dioxide as a Te source for MoTe_2_ synthesis. MoO_3_ was kept as a Mo source in all the syntheses of the MoX_2_ nanoparticles. Figure 7 above shows the schematic route taken for the synthesis of the nanoparticles.

### 3.2. Characterization

The morphology, crystallinity studies of the samples and particle size determination, was determined using a Bruker D8 X-ray diffractometer (Bruker, Madison, WI, USA). A Perkin-Elmer Lambda 25 UV–Vis spectrophotometer (Perkin Elmer, Boston, MA, USA) with the infrared data collected at 4000–300 cm^−1^ and a Perkin-Elmer Lambda 45 Fluorimeter (Perkin Elmer, Boston, MA, USA) at 250–800 nm were used to study the optical properties. Fourier-transform infrared spectroscopy was performed utilizing a FTIR-ATR Perkin-Elmer 4000 Hz Spectrophotometer (Perkin Elmer, Boston, MA, USA). The I–V photovoltaic measurements were conducted using a Keithley 2400 sourcemeter (Keithley, Cleveland, OH, USA). The developed quantum dot-sensitized solar cell which was utilized for the I–V measurements consisted of a synthesized MoX_2_ quantum dot as the sensitizer, a titanium dioxide photoanode with a 7.5 × 7.5 mm active area, a platinum counter electrode, and an Iodolyte redox mediator purchased from Solaronix (Solaronix, Aubonne, Switzerland). The quantum dot loading for sensitizing was performed using warm water, MoX_2_ nanoparticles, and coadsorbents (chenodeoxycholic acid). The Iodolyte redox mediator purchased from Solaronix was used with an iodide species with the molarity of 0.05 M. The thin film of TiO_2_ was then soaked in a photosensitized solution for 9 h. Platinum and TiO_2_ loaded with photosensitizer substrates were the intercalated ensuring electrical contacts. The electrolyte was then injected into the enclosed hollow on the left between the electrodes till the whole hollow was filled with the electrolyte. The power conversion efficiency (PCE) which measures how effectively the solar cell converts sunlight to electricity was calculated using Equation (2).

## 4. Conclusions

In conclusion, molybdenum chalcogenide nanoparticles were successfully synthesized using the colloidal bottom–up approach with the two-source precursor route. Successful synthesis was confirmed by the results shown when the nanoparticles were characterized. XRD results confirmed hexagonal arrangement of MoX_2_ and the size of all the MoX_2_ particles to be in the nanoparticle range, with MoS_2_ having the mean particle size of 1.1342 nm, MoSe_2_—0.7952 nm, MoTe_2_—0.8457 nm. FTIR analysis revealed the successful synthesis of MoX_2_ as vibrational bands were found at 812 cm^−1^, 689 cm^−1^, and 459 cm^−1^ for MoTe_2_, MoSe_2_, and MoS_2_, respectively. PL and UV–Vis revealed the optical properties of the Mo chalcogenides to be quantum-confined as observable peaks were noticed in all the MoX_2_ nanoparticles ranging from 302–322 nm confirming a blue shift from the red range which is usually around 600–700 nm. The application of the NPs synthesized in the QDSSCs was evaluated and the results showed that MoSe_2_ was the most efficient compound, with a PCE of 7.86%, followed by MoTe_2_ with 6.93% and MoS_2_ with 6.05%. Hence, it can be concluded that MoSe_2_ is the most efficient as a replacement for the dye in QDSSCs.

## Figures and Tables

**Table 1:** XRD parameters of the MoX_2_ nanoparticles.

Sample	2θ	Lattice Plane
MoS_2_	12.8^◦^, 24.2^◦^, 25.7^◦^, 27.5^◦^, 43.4^◦^	(002); (100); (102); (103); (006)
MoSe_2_	13.8^◦^, 32.2^◦^; 37.6^◦^; 53.8^◦^	(002); (100); (103); (110)
MoTe_2_	12.7^◦^, 27.3^◦^, 38.7^◦^, 43.1^◦^	(0002); (0004); (103); (0006)

**Table 2:** XRD parameters and the calculated mean sizes for the nanomaterials.

Sample	θ (degrees)	Mean FWHM (Å)	Calculated D (Å)
MoS_2_	12.9	1.3147	1.1342
MoSe_2_	16.04	1.8951	0.7952
MoTe_2_	13.3	1.7596	0.8457

**Table 3:** Grain size distribution for the MoX_2_ NPs calculated using SEM analysis.

Nanoparticles	Mean Particle Size (µm)
MoS_2_	1.122
MoSe_2_	0.81718
MoTe_2_	0.82577

**Table 4:** Photovoltaic parameters of the QDSSCs with the use of the MoX_2_ nanoparticles as the photoanode.

QDs	V_OC_ (mV)	I_SC_ (mA·cm^−2^)	FF (%)	PCE (%)
MoS_2_	0.68	12.6	70.6	6.05
MoSe_2_	0.72	14.8	73.7	7.86
MoTe_2_	0.70	14.4	68	6.93

## Data Availability

The data presented in the article are available upon request from the authors.

## Sample Availability

Samples of the compounds are available from the authors upon request.

## References

[1] Kalenga M.P., Govindraju S., Airo M., Moloto M.J., Sikhwivhilu L.M., Moloto N. (2015). Fabrication of a Schottky Device Using CuSe Nanoparticles: Colloidal versus Microwave Digestive Synthesis. J. Nanosci. Nanotechnol..

[2] Ewing R.C., Runde W., Albrecht-Schmitt T.E. (2010). Environmental impact of the nuclear fuel cycle: Fate of actinides. MRS Bull..

[3] Li G., Shrotriya V., Huang J., Yao Y., Moriarty T., Emery K., Yang Y. (2005). High-efficiency solution processable polymer photovoltaic cells by self-organization of polymer blends. Nat. Mater..

[4] Son D.-Y., Kim S.-G., Seo J.-Y., Lee S.-H., Shin H., Lee D., Park N.-G. (2018). Universal Approach toward Hysteresis-Free Perovskite Solar Cell via Defect Engineering. J. Am. Chem. Soc..

[5] Grätzel M. (2005). Solar Energy Conversion by Dye-Sensitized Photovoltaic Cells. Inorg. Chem..

[6] Abolhosseini S., Heshmati A., Altmann J. (2014). A Review of Renewable Energy Supply and Energy Efficiency Technologies.

[7] Kabashi S., Bekteshi S., Ahmetaj S., Kabashi G., Najdovski D., Zidansek A., Šlaus I. (2011). Effects of Kosovo’s energy use scenarios and associated gas emissions on its climate change and sustainable development. Appl. Energy.

[8] Beard M.C., Luther J.M., Nozik A.J., Sargent E.H. (2010). Multiple exciton generation in semiconductor quantum dots and electronically coupled quantum dot arrays for application to thirdgeneration photovoltaic solar cells. Colloid. Quantum Dot Optoelectron. Photovolt..

[9] Makinana S. (2018). Synthesis and characterization of CdSe quantum dots for solar cell applications. Master’s Dissertation.

[10] A Green M. (2002). Third generation photovoltaics: Solar cells for 2020 and beyond. Phys. E Low Dimens. Syst. Nanostruct..

[11] Hewa-Rahinduwage C.C., Geng X., Silva K.L., Niu X., Zhang L., Brock S.L., Luo L. (2020). Reversible Electrochemical Gelation of Metal Chalcogenide Quantum Dots. J. Am. Chem. Soc..

[12] Wu Y., Xu M., Chen X., Yang S., Wu H., Pan J., Xiong X. (2016). CTAB-assisted synthesis of novel ultrathin MoSe2nanosheets perpendicular to graphene for the adsorption and photodegradation of organic dyes under visible light. Nanoscale.

[13] Park J.C., Yun S.J., Kim H., Park J.-H., Chae S.H., An S.-J., Kim J.-G., Kim S.M., Kim K.K., Lee Y.H. (2015). Phase-Engineered Synthesis of Centimeter-Scale 1T′- and 2H-Molybdenum Ditelluride Thin Films. ACS Nano.

[14] Zhou J., Naiqin Z., Zhang X., Shi C., Liu E., Li J., Zhao N., Chunsheng S. (2015). 2D Space-Confined Synthesis of Few-Layer MoS2 Anchored on Carbon Nanosheet for Lithium-Ion Battery Anode. ACS Nano.

[15] Dar S.H., Thirumaran S., Selvanayagam S. (2015). Synthesis, spectral and X-ray structural studies on Hg(II) dithiocarbamate complexes: A new precursor for HgS nanoparticles. Polyhedron.

[16] Yang L., Cui X., Zhang J., Wang K., Shen M., Zeng S., Dayeh S., Feng L., Xiang B. (2015). Lattice strain effects on the optical properties of MoS2 nanosheets. Sci. Rep..

[17] Cao H., Wang H., Huang Y., Sun Y., Shi S., Tang M. (2016). Quantification of gold(III) in solution and with a test stripe via the quenching of the fluorescence of molybdenum disulfide quantum dots. Microchim. Acta.

[18] Amara K.K., Chu L., Kumar R., Toh M., Eda G. (2014). Wet chemical thinning of molybdenum disulfide down to its monolayer. APL Mater..

[19] Samadi M., Sarikhani N., Zirak M., Zhang H., Zhang H.-L., Moshfegh A.Z. (2018). Group 6 transition metal dichalcogenide nanomaterials: Synthesis, applications and future perspectives. Nanoscale Horiz..

[20] Lv R., Robinson J.A., Schaak R.E., Sun D., Sun Y., Mallouk T.E., Terrones M. (2015). Transition Metal Dichalcogenides and Beyond: Synthesis, Properties, and Applications of Single- and Few-Layer Nanosheets. Acc. Chem. Res..

[21] Gupta P., Rahman A.A., Subramanian S., Gupta S., Thamizhavel A., Orlova T., Rouvimov S., Vishwanath S., Protasenko V., Laskar M.R. (2016). Layered transition metal dichalcogenides: Promising near-lattice-matched substrates for GaN growth. Sci. Rep..

[22] Alavi M., Rahimi R., Maleki Z., Hosseini-Kharat M. (2020). Improvement of Power Conversion Efficiency of Quantum Dot-Sensitized Solar Cells by Doping of Manganese into a ZnS Passivation Layer and Cosensitization of Zinc-Porphyrin on a Modified Graphene Oxide/Nitrogen-Doped TiO_2_ Photoanode. ACS Omega.

[23] Lu S., Peng S., Zhang Z., Deng Y., Qin T., Huang J., Ma F., Hou J., Cao G. (2018). Impacts of Mn ion in ZnSe passivation on electronic band structure for high efficiency CdS/CdSe quantum dot solar cells. Dalton Trans..

[24] Agoro M.A., Meyer E.L., Mbese J., Manu K. (2020). Electrochemical Fingerprint of CuS-Hexagonal Chemistry from (Bis(N-1,4-Phenyl-N-(4-Morpholinedithiocarbamato) Copper(II) Complexes) as Photon Absorber in Quantum-Dot/Dye-Sensitised Solar Cells. Catalysts.

[25] Mbese J.Z., Meyer E.L., Agoro M.A. (2020). Electrochemical Performance of Photovoltaic Cells Using HDA Capped-SnS Nanocrystal from bis (N-1,4-Phenyl-N-Morpho-Dithiocarbamato) Sn(II) Complexes. Nanomaterials.

[26] Agoro M.A., Mbese J.Z., Meyer E.L. (2020). Electrochemistry of Inorganic OCT-PbS/HDA and OCT-PbS Photosensitizers Thermalized from Bis(N-diisopropyl-N-octyldithiocarbamato) Pb(II) Molecular Precursors. Molecules.

[27] Halimehjani A.Z., Torabi S., Amani V., Notash B., Saidi M.R. (2015). Synthesis and characterization of metal dithiocarbamate derivatives of 3-((pyridin-2-yl)methylamino)propanenitrile: Crystal structure of [3-((pyridin-2-yl)methylamino)propanenitrile dithiocarbamato] nickel(II). Polyhedron.

